# Comparative Transcriptomic Analysis Reveals Cultivar-Dependent Resistance Responses to *Pseudomonas tolaasii* in *Pleurotus ostreatus*

**DOI:** 10.3390/jof12070525

**Published:** 2026-07-17

**Authors:** Ja-Yoon Kim, Kang-Hyo Lee, Gyung-Sook Han, Gi-Hong An, Seong-Yeon Jo, Hye-Sung Park

**Affiliations:** Mushroom Research Division, National Institute of Horticultural and Herbal Science, Rural Development Administration, Eumseong 27709, Republic of Korea; xhfhcl@korea.kr (J.-Y.K.); agroup@korea.kr (K.-H.L.); kshan9@korea.kr (G.-S.H.); agiho@korea.kr (G.-H.A.); tjddus6325@korea.kr (S.-Y.J.)

**Keywords:** *Pleurotus ostreatus*, *Pseudomonas tolaasii*, brown blotch disease, transcriptome, disease resistance

## Abstract

*Pseudomonas tolaasii*, the causal agent of bacterial brown blotch disease, is a major threat to the yield and quality of oyster mushroom (*Pleurotus ostreatus*). To investigate the molecular basis of cultivar-dependent resistance, we performed comparative transcriptome analysis using the resistant cultivar ‘Aenutari’ (KMCC01220; AE) and the susceptible cultivar ‘Dahyun’ (KMCC04942; DH) under *P. tolaasii* challenge. The two cultivars showed contrasting phenotypic and transcriptomic responses after pathogen inoculation. DH exhibited visible disease symptoms and severe mycelial growth inhibition, with relative mycelial growth reduced to 33.97 ± 2.53% of the control, whereas AE maintained a relatively resistant phenotype and higher mycelial growth under pathogen challenge, reaching 51.19 ± 3.72% of the control. Consistent with these phenotypic differences, DH showed extensive transcriptional reprogramming, with 3585 up-regulated and 4377 down-regulated differentially expressed genes (DEGs). In contrast, AE showed a much narrower transcriptional response, with 181 up-regulated and 71 down-regulated DEGs. Functional enrichment analysis indicated that pathogen-responsive DEGs in AE were mainly associated with carbohydrate metabolism, extracellular regions, redox-related processes, and ion transport. In cultivar-wise comparisons under pathogen-inoculated conditions, genes highly expressed in AE were enriched in RNA splicing, mRNA processing, mitochondrial organization, mitochondrial gene expression, transmembrane transport, oxidoreductase activity, and sterol-related metabolism. These results indicate that the resistant phenotype of AE is associated with a more limited pathogen-induced transcriptional perturbation and the relative enrichment of gene modules related to RNA processing, mitochondrial organization and gene expression, membrane transport, redox-related processes, and sterol metabolism. This study provides a transcriptomic framework for understanding cultivar-dependent responses to bacterial brown blotch disease in oyster mushroom.

## 1. Introduction

*P. ostreatus*, commonly known as the oyster mushroom, is one of the most widely cultivated edible mushrooms worldwide and has substantial economic importance in the mushroom industry. In commercial mushroom production, both yield and external quality are directly linked to market value. Therefore, bacterial diseases that occur during cultivation are considered major limiting factors because they reduce not only fungal growth but also the visual quality and commercial acceptability of fruiting bodies. Among these diseases, brown blotch disease caused by *Pseudomonas tolaasii* is a representative bacterial disease that severely affects cultivated mushrooms, including *P. ostreatus* [1,2]. Bacterial blotch disease has been recognized as a persistent problem in mushroom production systems, and its incidence is strongly influenced by environmental conditions such as high humidity, condensation, and insufficient ventilation [3,4]. Infection by this pathogen results in visible discoloration, tissue degradation, and growth inhibition, leading to considerable losses in mushroom production [5,6,7]. Bacterial blotch can cause substantial production losses in commercial mushroom cultivation. Yield losses of up to 15% have been reported in Spanish mushroom crops, while a documented outbreak in commercial *Pleurotus eryngii* cultivation affected 39.75% of cultivated substrate blocks over a five-month period [1,3].

The pathogenicity of *P. tolaasii* has long been associated with the secretion of tolaasin, an extracellular lipodepsipeptide toxin. Tolaasin forms ion channels or pores in fungal cell membranes, disrupts osmotic balance and membrane integrity, and ultimately induces tissue collapse and brown blotch symptom development [8,9,10,11]. In addition to its pore-forming activity, tolaasin belongs to a broader group of bacterial lipodepsipeptides that interact with biological membranes and contribute to symptom development in cultivated mushrooms [12,13,14]. These findings indicate that brown blotch symptoms are not merely superficial discoloration but are closely linked to membrane damage and cellular dysfunction in mushroom tissues. Recent studies have further confirmed that tolaasin functions as a pore-forming bacterial peptide toxin and that its cytotoxicity is mediated through hydrophobic interactions and multimerization in host membranes [11]. Thus, membrane stability and cellular homeostasis are likely to be important biological components of the host response to *P. tolaasii* infection.

However, the pathogenesis of brown blotch disease cannot be fully explained by tolaasin activity alone. Recent studies have emphasized that bacterial blotch disease may involve taxonomically and phenotypically diverse bacterial populations, rather than a single uniform pathogen–toxin interaction [3,15,16]. Various pathogenic *Pseudomonas* strains have been isolated from diseased cultivated mushrooms, suggesting that bacterial blotch disease is caused by a complex interaction between pathogenic bacteria and mushroom hosts [7]. In addition, extracellular factors produced by *P. tolaasii*, including exopolysaccharides, have been reported to inhibit the growth of *P. ostreatus* mycelia [17]. These observations suggest that brown blotch disease should be understood as a complex pathosystem involving bacterial toxins, extracellular pathogenic factors, and host-dependent physiological responses, rather than as a disease caused by a single virulence factor. From the host perspective, resistance to brown blotch disease varies among *Pleurotus* genetic resources. Previous screening studies identified resistant, moderately resistant, and susceptible *P. ostreatus* strains against *P. tolaasii*, demonstrating that disease resistance is a detectable and exploitable trait at the cultivar or strain level [18]. Such cultivar-dependent variation provides a useful biological system for dissecting the molecular basis of mushroom defense responses. These findings also suggest that resistance is not simply determined by pathogen exposure but may be associated with cultivar-specific physiological and transcriptional regulatory programs.

Recent transcriptome-based studies have begun to reveal the molecular responses of edible mushrooms to bacterial infection. Comparative transcriptomic analyses of *P. ostreatus* and other cultivated mushrooms infected with *P. tolaasii* have shown that browning symptoms are accompanied by species- or strain-dependent transcriptional changes in genes related to cell wall-associated processes, oxidative responses, and defense-related regulation [6,19]. In particular, susceptible strains exhibited extensive cellular damage and broad transcriptional reprogramming, whereas resistant strains showed a more controlled response. These results suggest that the resistance phenotype may be associated not only with inducible defense responses but also with the ability to maintain cellular organization, redox balance, and stress-related regulatory networks during pathogen challenge. Nevertheless, the transcriptional modules that distinguish resistant and susceptible *P. ostreatus* cultivars during *P. tolaasii* infection remain insufficiently characterized. In fungi, stress responses are tightly connected with multiple cellular processes, including RNA processing, mitochondrial activity, redox homeostasis, membrane transport, and primary metabolism. Alternative splicing and mRNA processing have been proposed as important post-transcriptional regulatory mechanisms that contribute to fungal adaptation under environmental stress and host-interaction conditions [20,21,22]. Mitochondrial function is also closely associated with energy production, oxidative stress regulation, stress adaptation, and fungal pathogenicity or survival [23,24,25]. In addition, membrane-associated processes, including sterol and ergosterol metabolism, are essential for maintaining fungal membrane structure, rigidity, permeability, and membrane protein activity [26,27,28]. Because tolaasin directly targets membrane integrity, transcriptomic changes related to membrane homeostasis, mitochondrial function, and redox regulation may be particularly important for understanding the resistance response of *P. ostreatus* to *P. tolaasii*.

In this study, we investigated the physiological and transcriptomic responses of two *P. ostreatus* cultivars with contrasting disease phenotypes: the susceptible cultivar ‘Dahyun’ (DH) and the resistant cultivar ‘Aenutari’ (AE). We first compared phenotypic responses and mycelial growth dynamics after *P. tolaasii* inoculation. We then performed genome-wide RNA-seq analysis to identify differentially expressed genes and conducted GO and KEGG enrichment analyses to characterize pathogen-responsive and cultivar-dependent transcriptional programs. Finally, hierarchical clustering and cluster-based enrichment analyses were used to define major expression modules associated with resistance and susceptibility. Through this approach, we aimed to identify transcriptional pathways and functional modules associated with cultivar-dependent responses to *P. tolaasii* in *P. ostreatus*, with particular attention to RNA processing, mitochondria-associated processes, redox-related responses, membrane transport, and sterol-related metabolism.

## 2. Materials and Methods

### 2.1. Mushroom Materials, Pathogen Inoculation, and Dual Culture Assay

Two *P. ostreatus* cultivars with contrasting responses to bacterial brown blotch disease were used in this study: the susceptible cultivar ‘Dahyun’ (KMCC04942; DH) and the resistant cultivar ‘Aenutari’ (KMCC01220; AE). Fruiting bodies of both cultivars were cultivated at the Mushroom Research Division, National Institute of Horticultural and Herbal Science, Rural Development Administration, Eumseong, Republic of Korea. Fruiting bodies were produced under standard cultivation conditions at 16–18 °C, 85–95% relative humidity, and approximately 4500 ppm CO_2_. Mycelial cultures of both cultivars were grown on potato dextrose agar (PDA; MBcell, Seoul, Republic of Korea) at 25 °C in the dark for 7 days.

*P. tolaasii*, the causal agent of bacterial brown blotch disease, was cultured in R2B broth at 28 °C for 16 h with shaking. The bacterial suspension was adjusted to an optical density of OD_600_ = 0.1 using R2B broth before inoculation. For phenotypic analysis, 5 μL of the *P. tolaasii* suspension was applied to the pileus surface of fruiting bodies of DH and AE. Control samples were inoculated with 5 μL of R2B broth. After inoculation, the samples were incubated at 25 °C and 90% relative humidity in the dark. Disease symptoms, including discoloration, visible infection areas, and local tissue degradation, were observed and recorded using a digital camera at 24 h post-inoculation. Sixteen biological replicates were used for each treatment. A formal a priori power analysis was not performed; the replicate number was selected to provide sufficient representation of biological variation among individual fruiting bodies and to allow consistent evaluation of visible disease symptoms.

To compare the effect of *P. tolaasii* on mycelial growth, a dual culture assay was performed on PDA plates. A 5-mm mycelial plug of each cultivar was placed on a PDA plate, and *P. tolaasii* was inoculated 2 cm away from the fungal mycelial plug. Control plates were prepared without pathogen inoculation. Plates were incubated at 25 °C in the dark for 7 days. Mycelial growth was quantified by measuring the colony area from plate images using ImageJ software version 1.54k. Relative mycelial growth was calculated as the percentage of the mycelial area in the *P. tolaasii*-inoculated treatment relative to that of the corresponding non-inoculated control. Five independent biological replicates were used for each cultivar.

### 2.2. RNA Extraction, Library Construction, and Sequencing

For RNA-seq analysis, four experimental groups were prepared with three biological replicates per group: mock-treated DH (C_DH), *P. tolaasii*-inoculated DH (I_DH), mock-treated AE (C_AE), and *P. tolaasii*-inoculated AE (I_AE). Fruiting-body tissues for RNA-seq analysis were collected at 24 h post-inoculation. Collected samples were stored at −80 °C until RNA extraction. Frozen samples were ground in liquid nitrogen, and total RNA was extracted from approximately 100 mg of tissue using TRIzol reagent (Invitrogen, CA, USA). Genomic DNA contamination was removed by DNase I treatment (QIAGEN). Total RNA concentration was measured using Quant-IT RiboGreen (Invitrogen), and RNA quality was examined using TapeStation RNA ScreenTape (Agilent Technologies, Santa Clara, CA, USA). Only RNA samples with an RNA integrity number (RIN) ≥ 6.0 were used for library construction. For each sample, 1 μg of total RNA was used to construct an RNA-seq library using the NEXTFLEX^®^ Rapid Directional RNA-Seq Kit 2.0 (PerkinElmer Inc., Waltham, MA, USA). Briefly, poly(A)-containing mRNA was selectively enriched using poly-T beads and fragmented to an appropriate size. The fragmented RNA was synthesized into first-strand cDNA using reverse transcriptase and primers, followed by second-strand cDNA synthesis. After A-tailing and adapter ligation, final cDNA libraries were generated. Library quality was assessed using TapeStation D1000 ScreenTape (Agilent Technologies), and libraries that passed quality control were sequenced on an Illumina NovaSeq platform using paired-end sequencing with 2 × 151 bp reads.

### 2.3. RNA-Seq Data Processing and Functional Analysis

Raw reads were processed using Trimmomatic v0.39 to remove adapter sequences and low-quality bases [29]. Quality trimming was performed using the SLIDINGWINDOW, LEADING, and TRAILING options with the following criteria: window size = 4, mean quality ≥ 15, LEADING and TRAILING ≥ 3, and minimum read length ≥ 36 bp. The average trimmed/raw ratio was 97.55%, indicating that most sequencing reads were retained after quality filtering. Cleaned reads were mapped to the *P. ostreatus* reference genome obtained from NCBI (assembly accession: GCA_947034855.1) [30], which consists of 12 chromosomes and two organellar sequences, using Bowtie2 v2.4.1 [31]. According to the analysis report, Bowtie2 v2.4.1 was used for mapping with a mismatch threshold of ≤2 bp, and gene-level read counts were obtained by counting reads mapped to each annotated gene. Across the 12 RNA-seq libraries, mapping rates to the *P. ostreatus* reference genome ranged from 61.80% to 77.10%, with an average mapping rate of 72.35% (Table S1). Gene expression values were normalized using the DESeq package in R version 4.3.2 [32]. Gene annotation was performed by BLASTX against the NCBI NR Fungi database with an e-value threshold of ≤1 × 10^−10^.

Principal component analysis (PCA) was performed using normalized count data to evaluate the reproducibility of biological replicates and transcriptomic variation among samples. Differentially expressed genes (DEGs) were identified from both inoculated versus mock-treated comparisons and cultivar-wise comparisons. The inoculated versus mock-treated comparisons included I_DH vs. C_DH and I_AE vs. C_AE, whereas the cultivar-wise comparisons included C_AE vs. C_DH and I_AE vs. I_DH. DEGs were selected using the criteria of |log_2_(fold change)| ≥ 1 and false discovery rate (FDR) ≤ 0.01. Genes with log_2_(fold change) > 1 were defined as up-regulated DEGs, whereas genes with log_2_(fold change) < −1 were defined as down-regulated DEGs. Hierarchical clustering was performed based on the log_2_ fold-change profiles of DEGs across the relevant pairwise comparisons. Pairwise distances between genes were calculated using Euclidean distance, and hierarchical agglomerative clustering was performed using the complete-linkage method. To determine the appropriate number of clusters, silhouette analysis was performed across candidate cluster numbers, and six clusters were selected because this solution provided a favorable balance between cluster separation and biological interpretability. The resulting six clusters were used to summarize the major expression patterns and to facilitate cluster-wise functional enrichment analysis.

Gene Ontology (GO) enrichment analysis was performed to identify biological processes, cellular components, and molecular functions associated with DEGs [33]. GO enrichment analysis was performed using the annotated gene set as the background, and enriched GO terms were identified using Fisher’s exact test. Raw *p*-values from the GO enrichment analysis were adjusted for multiple testing using the false discovery rate (FDR) method, and GO terms with an FDR-adjusted *p*-value < 0.05 were considered significantly enriched. KEGG pathway analysis was performed by BLASTP searches against KEGG amino acid sequences using an e-value threshold of ≤1 × 10^−10^ and best-hit annotation [34]. For KEGG pathway enrichment analysis, raw *p*-values were adjusted for multiple testing using the Benjamini–Hochberg procedure, and pathways with an FDR-adjusted *p*-value < 0.05 were considered significantly enriched.

### 2.4. qRT-PCR Validation

To validate the RNA-seq results, selected representative DEGs were analyzed by quantitative real-time PCR (qRT-PCR). Candidate genes were selected from the I_AE vs. I_DH comparison based on their differential expression patterns and relevance to the major functional modules identified by RNA-seq analysis. Total RNA samples used for RNA-seq analysis were also used for qRT-PCR validation. First-strand cDNA was synthesized from total RNA using the HelixCript™ Easy cDNA Synthesis Kit (NanoHelix Co., Ltd., Daejeon, Republic of Korea) according to the manufacturer’s instructions. qRT-PCR was performed using AccuPower^®^ 2X GreenStar™ qPCR Master Mix (Bioneer, Daejeon, Republic of Korea) using a Thermal Cycler Dice^®^ Real Time System III (Takara Bio Inc., Shiga, Japan). Relative gene expression levels were calculated using the 2^−ΔΔCt^ method, and expression values were normalized against the *P. ostreatus* actin gene. Three biological replicates were used for each sample group, and primer sequences used for qRT-PCR are listed in Table S2.

### 2.5. Statistical Analysis

All quantitative data are presented as mean ± SD unless otherwise indicated. Statistical significance between two groups was determined using Student’s *t*-test. Differences were considered statistically significant at *p* < 0.05 and highly significant at *p* < 0.01. For transcriptome analysis, DEGs were identified using |log_2_(fold change)| ≥ 1 and FDR ≤ 0.01. For functional enrichment analyses, GO terms and KEGG pathways with FDR-adjusted *p*-values < 0.05 were considered significantly enriched. Agreement between RNA-seq- and qRT-PCR-derived log_2_ fold-change values was assessed using Pearson correlation analysis and linear regression.

## 3. Results

### 3.1. Phenotypic Observation and In Vitro Mycelial Growth Dynamics

**Figure 1:**
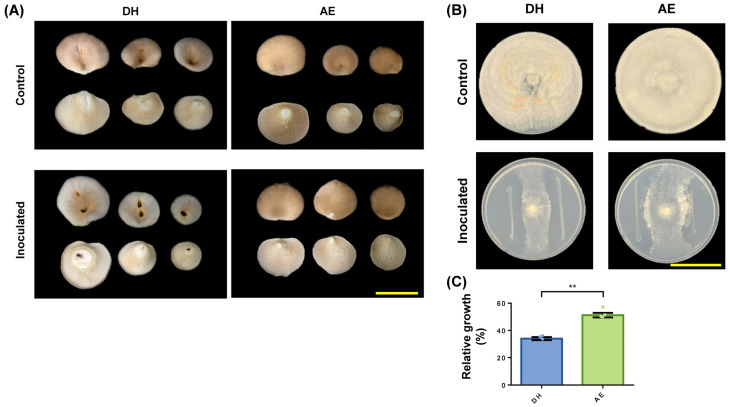
Phenotypic and mycelial growth responses of *Pleurotus ostreatus* cultivars to *Pseudomonas tolaasii* inoculation. (**A**) Representative phenotypic images of ‘Dahyun’ (DH, susceptible) and ‘Aenutari’ (AE, resistant) under R2B-treated control and *P. tolaasii*-inoculated conditions at 24 h post-inoculation. Scale bar = 4 cm. (**B**) Representative images of the dual culture assay between each cultivar and *P. tolaasii*. (**C**) Relative mycelial growth of DH and AE after 7 days of dual culture, calculated from ImageJ-based measurements of mycelial colony area and expressed relative to the corresponding non-inoculated control. Data are presented as mean ± SD from five independent biological replicates. Asterisks indicate a significant difference in relative mycelial growth between *P. tolaasii*-inoculated DH and AE, as determined by Student’s *t*-test (** *p* < 0.01), not comparisons between inoculated and non-inoculated controls within each cultivar. Scale bar = 1 cm.

To compare the resistance responses of the two mushroom cultivars, we first examined the phenotypes of the susceptible cultivar DH and the resistant cultivar AE after pathogen inoculation. At 24 h post-inoculation, DH developed visible infection areas accompanied by localized tissue degradation, whereas AE maintained a phenotype largely comparable to that of the mock-treated control (Figure 1A and Figure S1). These contrasting symptoms confirmed a clear cultivar-dependent difference in disease response at the fruiting-body level.

These host–pathogen interactions were further examined at the mycelial level using a dual-culture assay on PDA plates (Figure 1B). After 7 days of co-culture, *P. tolaasii* inhibited mycelial expansion in both cultivars, although the extent of growth inhibition differed markedly between DH and AE. Quantification of colony area showed that relative mycelial growth was significantly lower in DH, reaching 33.97 ± 2.53% of the corresponding non-inoculated control, whereas AE maintained 51.19 ± 3.72% of the control (Figure 1C). The stronger reduction in DH indicates a greater degree of pathogen-associated growth inhibition, whereas AE retained a larger proportion of its control growth under the same challenge conditions. The difference in relative mycelial growth between the two cultivars was highly significant (*p* < 0.01). Together with the contrasting fruiting-body phenotypes, these results show that DH and AE differ consistently in their responses to *P. tolaasii* at both fruiting-body and mycelial growth levels, providing a phenotypic basis for the subsequent comparative transcriptomic analyses.

### 3.2. Global Transcriptomic Profiling and Identification of DEGs

**Figure 2:**
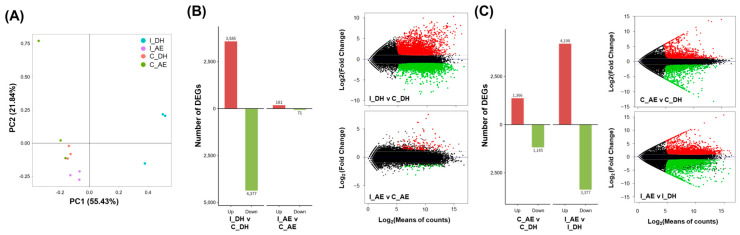
PCA and DEG analysis of *P. ostreatus* cultivars in response to *P. tolaasii* inoculation. (**A**) Principal component analysis based on normalized gene expression counts from four experimental groups: C_DH, mock-treated ‘Dahyun’; I_DH, *P. tolaasii*-inoculated ‘Dahyun’; C_AE, mock-treated ‘Aenutari’; and I_AE, *P. tolaasii*-inoculated ‘Aenutari’. Each point represents one biological replicate (*n* = 3). (**B**,**C**) Bar plots and MA plots showing DEGs from treatment-wise comparisons ((**B**); I_DH vs. C_DH and I_AE vs. C_AE) and cultivar-wise comparisons ((**C**); C_AE vs. C_DH and I_AE vs. I_DH). DEGs were defined by |log_2_(fold change)| ≥ 1 and FDR < 0.01.

To investigate the global transcriptomic variation underlying the distinct responses of the two cultivars, principal component analysis (PCA) was performed using the RNA-seq datasets. PC1 and PC2 accounted for 55.43% and 21.84% of the total variance, respectively. Biological replicates generally grouped according to their experimental conditions, although one C_AE replicate showed greater separation from the other C_AE replicates along PC2 (Figure 2A). Despite this within-group variation, the four experimental groups remained distinguishable in the PCA space, supporting subsequent comparative transcriptomic analyses.

Differentially expressed genes (DEGs) were identified using the criteria of |log_2_(fold change)| ≥ 1 and an FDR < 0.01 (Table S3). Upon pathogen inoculation, DH showed extensive transcriptomic changes, with 3585 up-regulated and 4377 down-regulated genes compared with its mock control (Figure 2B and Figure S2). In contrast, AE showed a narrower inoculation-induced response, with 181 up-regulated and 71 down-regulated genes under the same condition. In addition to these treatment-wise responses, cultivar-wise comparisons revealed clear transcriptional divergence between DH and AE. Under mock-treated conditions, the C_AE vs. C_DH comparison identified 1366 AE-enriched and 1185 DH-enriched genes, indicating that the two cultivars possess distinct basal expression profiles (Figure 2C and Figure S2). This cultivar-dependent divergence became more pronounced after pathogen inoculation, with 4198 AE-enriched and 3377 DH-enriched genes in the I_AE vs. I_DH comparison. Thus, the RNA-seq dataset captured two layers of cultivar-dependent variation: pre-existing transcriptional differences between DH and AE and distinct pathogen-induced transcriptional responses within each cultivar. The strong transcriptional shift in DH contrasted with the more restricted response in AE, suggesting that the resistant phenotype of AE was accompanied by a lower degree of pathogen-induced transcriptomic perturbation from its basal state.

### 3.3. Functional Enrichment and Clustering Analysis of Pathogen-Responsive DEGs

**Figure 3:**
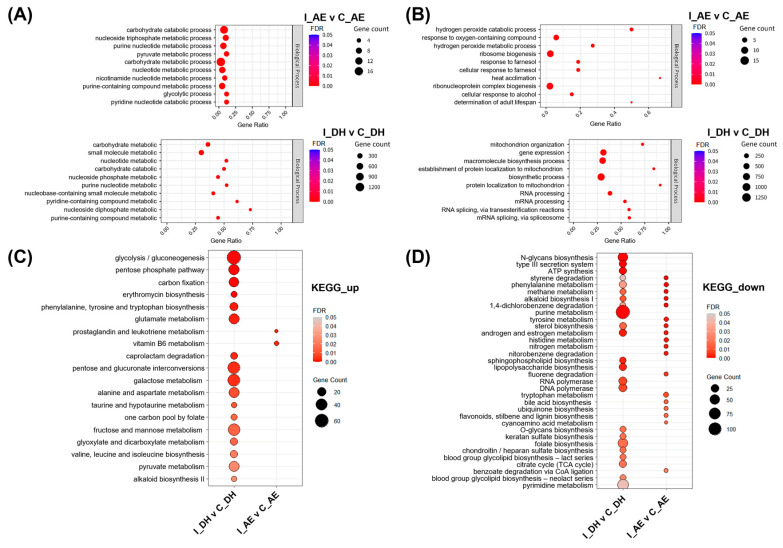
Functional enrichment analysis of treatment-responsive DEGs in DH and AE after *P. tolaasii* inoculation. (**A**,**B**) Gene Ontology (GO) enrichment bubble plots for up-regulated (**A**) and down-regulated (**B**) DEGs identified from treatment-wise comparisons in DH and AE. Treatment-wise comparisons included I_DH vs. C_DH and I_AE vs. C_AE. (**C**,**D**) KEGG pathway enrichment bubble plots for up-regulated (**C**) and down-regulated (**D**) DEGs from the same treatment-wise comparisons. Major significantly enriched KEGG pathways were associated with carbohydrate and central carbon metabolism, amino acid metabolism, nucleotide metabolism, lipid-related metabolism, glycan biosynthesis and metabolism, energy metabolism, and metabolism of cofactors and vitamins. For GO enrichment plots, the *x*-axis represents the GeneRatio and the *y*-axis indicates enriched GO terms. For KEGG enrichment plots, the *x*-axis indicates the pairwise comparisons and the *y*-axis represents significantly enriched KEGG pathways. Bubble size represents the number of DEGs assigned to each GO term or KEGG pathway, and color intensity represents the FDR-adjusted *p*-value. GO terms and KEGG pathways with FDR < 0.05 were considered significantly enriched.

To characterize the transcriptional responses induced by pathogen inoculation in each cultivar, GO and KEGG enrichment analyses were performed using DEGs identified from the inoculated versus mock-treated comparisons (Table S3). In AE, up-regulated DEGs were mainly enriched in carbohydrate metabolic process, carbohydrate catabolic process, glycolytic process, pyruvate metabolic process, extracellular region, extracellular exosome, glyceraldehyde-3-phosphate dehydrogenase activity, nucleoside diphosphate kinase activity, and xylanase activity (Figure 3A). In contrast, down-regulated DEGs in AE were associated with reactive oxygen species metabolic process, rRNA processing, calcium ion transport, ribonucleoprotein complex, nucleolus, peroxiredoxin activity, and calcium ion transmembrane transporter activity (Figure 3B). In DH, pathogen inoculation induced broader GO enrichment patterns than in AE. Up-regulated DEGs in DH were enriched in carbohydrate metabolic process, nucleotide metabolic process, small molecule metabolic process, cytosol, extracellular region, catalytic activity, oxidoreductase activity, and hydrolase activity (Figure 3A). Down-regulated DEGs in DH were mainly associated with mitochondrion organization, gene expression, macromolecule biosynthetic process, RNA processing, mRNA processing, RNA splicing, mitochondrial components, and transmembrane transporter activity (Figure 3B).

KEGG enrichment analysis showed that pathogen-responsive DEGs in DH were significantly enriched across a broader range of metabolic pathways than those in AE, particularly among the up-regulated DEGs (Figure 3C,D). In DH, significant enrichment was observed in pathways associated with central carbon and carbohydrate metabolism, including glycolysis/gluconeogenesis, the pentose phosphate pathway, pyruvate metabolism, galactose metabolism, and fructose and mannose metabolism, together with several amino acid- and nucleotide-related pathways. In contrast, only a limited number of KEGG pathways remained significantly enriched among the up-regulated DEGs in AE, whereas its down-regulated DEGs were associated with a more diverse but comparatively limited set of metabolic pathways. Overall, the FDR-adjusted KEGG results support broader pathogen-induced metabolic reprogramming in DH and a more restricted transcriptional response in AE.

To further examine pathogen-responsive expression patterns, hierarchical clustering was performed using DEGs from the I_DH vs. C_DH and I_AE vs. C_AE comparisons. A total of 8013 DEGs were divided into six clusters, containing 1858, 3567, 1739, 814, 24, and 11 genes in clusters 1–6, respectively (Figure S3). GO enrichment analysis of these clusters showed that the major expression modules were associated with primary metabolism, nucleotide and carbohydrate metabolism, RNA processing, mitochondrial function, membrane trafficking, lipid-related processes, and redox-associated enzyme activity (Figure S4). Overall, these results indicate that pathogen inoculation caused extensive transcriptomic reprogramming in DH, whereas AE showed a more limited but functionally distinct response involving metabolic, extracellular, redox, and ion transport-related processes.

### 3.4. Cultivar-Wise DEG Clustering Reveals Distinct Functional Modules Between DH and AE

**Figure 4:**
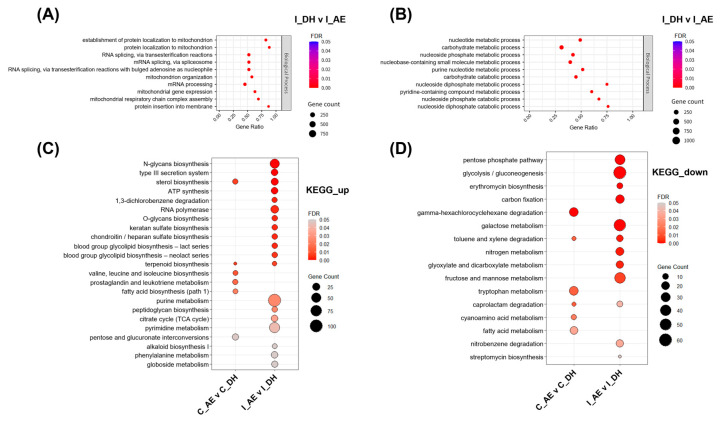
Functional enrichment analysis of cultivar-wise DEGs between AE and DH under *P. tolaasii*-inoculated conditions. (**A**,**B**) Gene Ontology (GO) enrichment bubble plots for up-regulated (**A**) and down-regulated (**B**) DEGs identified from the I_AE vs. I_DH comparison. Up-regulated DEGs indicate genes expressed at higher levels in I_AE than in I_DH, whereas down-regulated DEGs indicate genes expressed at lower levels in I_AE than in I_DH. (**C**,**D**) KEGG pathway enrichment bubble plots for up-regulated (**C**) and down-regulated (**D**) DEGs from the same cultivar-wise comparison. Major significantly enriched KEGG pathways included those related to glycan biosynthesis and metabolism, sterol and lipid-related metabolism, energy metabolism, nucleotide metabolism, and central carbon and carbohydrate metabolism. For GO enrichment plots, the *x*-axis represents the GeneRatio and the *y*-axis indicates enriched GO terms. For KEGG enrichment plots, the *x*-axis indicates the pairwise comparisons and the *y*-axis represents significantly enriched KEGG pathways. Bubble size represents the number of DEGs assigned to each GO term or KEGG pathway, and color intensity represents the FDR-adjusted *p*-value. GO terms and KEGG pathways with FDR < 0.05 were considered significantly enriched.

To identify transcriptional differences associated with the contrasting resistance phenotypes, GO and KEGG enrichment analyses were performed using DEGs identified from the I_AE vs. I_DH comparison under pathogen-inoculated conditions. Genes up-regulated in AE relative to DH were strongly enriched in RNA splicing, mRNA processing, protein localization to mitochondrion, mitochondrial organization, mitochondrial gene expression, mitochondrial respiratory chain complex assembly, transmembrane transporter activity, and oxidoreductase activity (Figure 4A). By contrast, genes down-regulated in AE relative to DH were mainly associated with carbohydrate metabolic process, nucleotide metabolic process, carbohydrate catabolic process, cytosol, cytoplasm, oxidoreductase activity, and amylase activity (Figure 4B). KEGG analysis further revealed distinct cultivar-dependent metabolic patterns (Figure 4C,D). DEGs expressed at higher levels in AE than in DH were significantly enriched in pathways related to glycan biosynthesis, sterol biosynthesis, ATP synthesis, RNA polymerase, purine and pyrimidine metabolism, and the citrate cycle (TCA cycle). In contrast, DEGs expressed at lower levels in AE than in DH, corresponding to genes relatively enriched in DH, were predominantly associated with central carbon and carbohydrate metabolism, including the pentose phosphate pathway, glycolysis/gluconeogenesis, carbon fixation, galactose metabolism, glyoxylate and dicarboxylate metabolism, and fructose and mannose metabolism.

**Figure 5:**
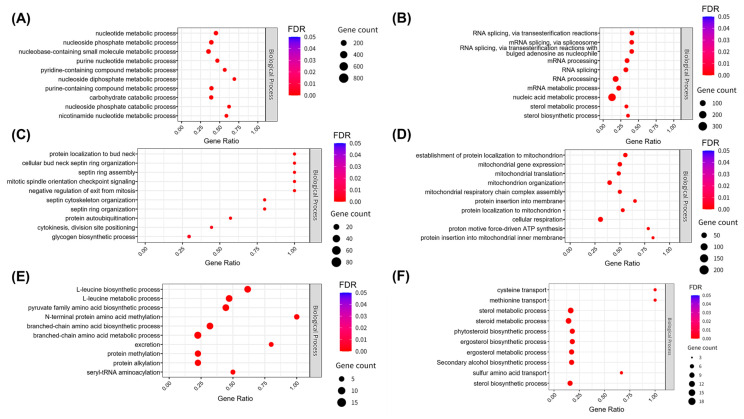
Functional enrichment analysis of gene clusters derived from cultivar-wise comparisons between DH and AE. (**A**–**F**) Bubble plots showing significantly enriched Gene Ontology (GO) terms for the six major clusters identified from cultivar-wise DEG clustering. Each panel corresponds to (**A**) Cluster 1, (**B**) Cluster 2, (**C**) Cluster 3, (**D**) Cluster 4, (**E**) Cluster 5, and (**F**) Cluster 6. The *x*-axis represents the enrichment factor, and the *y*-axis indicates enriched GO terms within biological process, cellular component, and molecular function categories. Bubble size is proportional to the number of DEGs assigned to each term, and color intensity indicates statistical significance (FDR < 0.05).

Cluster-based enrichment analysis further resolved the cultivar-dependent transcriptional modules. A total of 8456 DEGs were separated into six clusters, containing 2482, 1807, 1019, 2164, 357, and 627 genes in clusters 1–6, respectively (Figure S5). GO analysis showed that cluster 1 was mainly associated with carbohydrate and nucleotide metabolism, cytosol, oxidoreductase activity, and ligase activity, whereas clusters 2 and 4 were enriched in RNA splicing, mRNA processing, mitochondrial gene expression, mitochondrial translation, cellular respiration, and ATP synthesis-related processes (Figure 5). Clusters 3, 5, and 6 were associated with cytokinesis-related processes, glycogen biosynthesis, branched-chain amino acid metabolism, protein methylation, sulfur amino acid transport, methionine transport, and sterol biosynthesis. Collectively, the cultivar-wise enrichment and clustering analyses show that AE and DH activate distinct transcriptional programs following pathogen inoculation.

To further examine representative genes underlying the cultivar-dependent expression patterns, qRT-PCR analysis was performed using 12 selected DEGs from the I_AE vs. I_DH comparison. Seven AE-enriched genes (ENSOPET00005000131, ENSOPET00005000137, ENSOPET00005002717, ENSOPET00005012956, ENSOPET00005012971, ENSOPET00005025345, and ENSOPET00005031318) and five DH-enriched genes (ENSOPET00005009458, ENSOPET00005012618, ENSOPET00005013427, ENSOPET00005021531, and ENSOPET00005025498) were selected from major functional modules identified by the cultivar-wise transcriptome and clustering analyses (Figure S6). These candidates represented mitochondrial function, redox-related responses, sterol metabolism, carbohydrate and energy metabolism, amino acid and nitrogen metabolism, and nucleotide metabolism (Table S4). Among the AE-enriched candidates, ENSOPET00005002717 was annotated as the mitochondrial protein transporter Tim10, ENSOPET00005012956 as cTPxI, and ENSOPET00005031318 as delta(24)-sterol C-methyltransferase. Their expression patterns were consistent with the enrichment of mitochondria-associated, redox-related, and sterol metabolism modules in AE. The qRT-PCR expression patterns were generally consistent with the RNA-seq data, and linear regression analysis showed a strong correlation between RNA-seq- and qRT-PCR-derived fold changes (R = 0.854; Figure S6C). Together, these representative genes provide gene-level support for the contrasting transcriptional patterns observed between AE and DH and define a focused set of cultivar-associated candidates for further functional characterization.

## 4. Discussion

Brown blotch disease in *P. ostreatus* can be viewed as a cultivar-dependent outcome of host responses to bacterial virulence factors [4]. In this study, DH showed stronger disease-associated growth inhibition, whereas AE maintained a comparatively resistant phenotype after *P. tolaasii* inoculation (Figure 1). The transcriptomic responses also differed markedly between cultivars: DH underwent broad transcriptional reprogramming, whereas AE showed a substantially more restricted response (Figure 2). This contrast suggests that resistance is not simply related to the magnitude of inducible DEG accumulation. Instead, the AE response may reflect a less extensively perturbed transcriptional state under bacterial challenge, consistent with previous reports of cultivar- and strain-dependent variation in brown blotch susceptibility in *Pleurotus* mushrooms [5,6,18].

The two cultivars also differed in their basal transcriptional states (Figure 2C). Under mock-treated conditions, the C_AE vs. C_DH comparison revealed clear cultivar-dependent expression differences, indicating that AE and DH were transcriptionally distinct before pathogen exposure. Following inoculation, the two cultivars differed further in the extent of transcriptional change from their respective basal states. Thus, the AE phenotype appears to be associated with both a distinct basal expression program and selective transcriptional adjustment during bacterial challenge, rather than with the absolute number of DEGs alone. Because transcriptomes were analyzed at a single post-inoculation stage, the observed DEG amplitudes may also reflect cultivar-dependent differences in the timing or intensity of pathogen perception and disease progression.

The biological relevance of this cultivar-dependent response is closely related to the membrane-targeting activity of *P. tolaasii*. Tolaasin, a structurally defined lipodepsipeptide toxin, induces brown blotch symptoms through ion channel formation and pore-generating activity on host membranes [8,9,10]. Its cytotoxicity depends on hydrophobic interactions and multimerization within membranes, whereas tolaasin inhibitory factors suppress both cytotoxicity and disease severity [11,35]. However, brown blotch pathogenesis is unlikely to be explained by tolaasin alone. Recent studies have further emphasized that blotch-like symptoms in cultivated mushrooms can involve diverse bacterial pathogens and distinct toxin-related mechanisms [36]. Multiple pathogenic *Pseudomonas* lineages can induce blotch-like symptoms, and *P. tolaasii* pathogenicity involves additional virulence factors, including exopolysaccharides that directly inhibit *P. ostreatus* mycelial growth [7,13,16,17,37]. Thus, the host transcriptome likely reflects a combined stress state involving membrane pore formation, ion disequilibrium, impaired surface integrity, and extracellular inhibitory effects.

The host transcriptional modules identified in this study can be interpreted in relation to these known pathogenic properties of *P. tolaasii*. Tolaasin-mediated pore formation is expected to impose membrane-centered stress, including altered ion balance, impaired membrane integrity, and secondary oxidative perturbation. Consistent with this mode of action, cultivar-wise enrichment analysis showed that AE-associated genes were enriched in transmembrane transporter activity, sterol/ergosterol biosynthesis, oxidoreductase activity, mitochondrial organization, and mitochondrial gene expression (Figure 4 and Figure 5). These functional categories do not directly measure toxin activity, but they correspond to cellular processes that are closely connected with membrane organization, redox balance, and energy-associated metabolism. In addition, the reported inhibitory effects of *P. tolaasii* exopolysaccharides on *P. ostreatus* mycelial growth provide a possible context for the extracellular-region and carbohydrate metabolism-related responses observed after inoculation (Figure 3). Therefore, the cultivar-dependent transcriptome appears to reflect host responses to multiple layers of bacterial stress, including membrane disruption, extracellular inhibition, ion disequilibrium, and downstream metabolic disturbance. These transcriptional responses are consistent with the known pathogenic activities of *P. tolaasii*, although the specific relationships between individual bacterial virulence activities and host transcriptional modules remain unresolved.

Functional enrichment analyses further suggest that the AE response is characterized by a more restricted and functionally differentiated transcriptional pattern rather than broad transcriptional reprogramming. Treatment- and cultivar-wise enrichment analyses showed that the two cultivars activated distinct biological programs after pathogen inoculation (Figure 3 and Figure 4). Cluster-based analysis revealed that AE-associated clusters were enriched for RNA splicing, mRNA processing, mitochondrial gene expression, mitochondrial translation, respiration, ATP synthesis, sulfur amino acid transport, methionine transport, and sterol/ergosterol biosynthesis (Figure 5). These functional categories may represent transcriptional modules associated with membrane-related processes, energy-associated metabolism, and post-transcriptional regulation. Ergosterol is essential for fungal membrane integrity and organization, and changes in sterol composition broadly influence membrane function, stress responses, morphogenesis, and antifungal susceptibility [26,27,28]. In addition, ABC and MFS transporters function as broad stress-response components that support fungal adaptation to hostile environments [38]. Therefore, the enrichment of sterol-related metabolism and transporter-associated genes in AE identifies these processes as candidate components of the cultivar-dependent transcriptional response to membrane-targeting bacterial stress.

The mitochondrial and RNA-processing signatures of AE also provide important insight into the cultivar-dependent response. Fungal mitochondria are central regulators of respiration, oxidative-stress homeostasis, metabolic adaptation, and stress-associated fitness [23,24]. The enrichment of mitochondrial organization, respiratory-chain complex assembly, and ATP synthesis-related functions in AE indicates a transcriptional bias toward mitochondria-associated processes in the resistant cultivar under bacterial stress. These patterns are consistent with differential regulation of energy-associated cellular processes between the two cultivars, although their functional consequences remain to be determined. In parallel, the enrichment of RNA splicing- and mRNA processing-related modules indicates differential transcriptional regulation of RNA-associated processes in AE. Although alternative splicing is an important regulatory layer in fungal stress adaptation, whether these expression patterns are accompanied by changes in splicing events remains unresolved [20,22].

By contrast, the susceptible DH cultivar showed a much larger DEG burden together with more severe disease symptoms (Figure 1 and Figure S1). In particular, carbohydrate metabolism-related genes showed comparison-dependent expression patterns: these genes were induced in AE after pathogen inoculation, but their expression levels were lower in I_AE than in I_DH in the cultivar-wise comparison (Figure 3 and Figure 4). This apparent discrepancy reflects the relative nature of DEG analysis rather than a true contradiction. The higher expression of carbohydrate metabolism-related genes in DH suggests that the susceptible cultivar underwent broader metabolic reprogramming under pathogen-induced stress. Thus, the more extensive transcriptional activation of carbohydrate metabolism in DH is consistent with a greater degree of metabolic perturbation, whereas the more moderate response in AE may reflect a comparatively limited transcriptional shift. The susceptible phenotype may therefore be associated with broader disruption of cellular processes under bacterial challenge, rather than simply with differences in pathogen perception.

Taken together, the data support a transcriptomic model in which cultivar-dependent responses to *P. tolaasii* differ in both the extent and functional composition of transcriptional reprogramming. AE displayed a restricted response associated with sterol-related, transport, mitochondria-associated, sulfur amino acid transport, and RNA-processing functions, whereas DH showed broader transcriptional changes accompanying more severe disease symptoms. These findings provide candidate molecular features for further investigation of brown blotch resistance in *P. ostreatus*. Further physiological, biochemical, and functional analyses will help clarify their contributions to cultivar-dependent resistance to *P. tolaasii*.

## Data Availability

Raw mRNA-seq data have been deposited in the NCBI Sequence Read Archive (SRA) under BioProject accession number PRJNA1482220.

## Supplementary Materials

The following supporting information can be downloaded at: https://www.mdpi.com/article/10.3390/jof12070525/s1, Figure S1. Comparative phenotypic responses of ‘Dahyun’ and ‘Aenutari’ to *P. tolaasii* inoculation. Representative phenotypic images of the susceptible cultivar ‘Dahyun’ (DH) and the resistant cultivar ‘Aenutari’ (AE) under R2B-treated control and *P. tolaasii*-inoculated conditions at 24 h post-inoculation. Scale bar = 4 cm. Figure S2. Volcano plots of differentially expressed genes in treatment-wise and cultivar-wise comparisons. Volcano plots showing DEGs from the treatment-wise comparisons, including I_DH vs. C_DH and I_AE vs. C_AE, cultivar-wise comparisons, including C_AE vs. C_DH and I_AE vs. I_DH. The *x*-axis represents log_2_ fold change, and the *y*-axis represents the −log_10_-transformed FDR-adjusted *p*-value. Red and green points indicate significantly up-regulated and down-regulated DEGs, respectively, whereas black points represent genes that did not meet the DEG criteria. Vertical dashed lines indicate log_2_ fold-change thresholds of ±1, and the horizontal dashed line indicates an FDR threshold of 0.01. DEGs were defined by |log_2_(fold change)| ≥ 1 and FDR < 0.01. Figure S3. Hierarchical clustering and expression profiles of DEGs from treatment-wise comparisons. (A) Hierarchical clustering heatmap of DEGs identified from treatment-wise comparisons, including I_DH vs. C_DH and I_AE vs. C_AE. Each row represents a gene, and columns represent biological replicates (*n* = 3) of the four experimental groups. The color scale indicates log_2_ fold change, with red indicating positive values, green indicating negative values, and black indicating little or no change. (B) Expression profiles of the six major clusters. The *x*-axis represents the pairwise comparisons, and the *y*-axis represents log_2_ fold change. Gray lines represent the expression patterns of individual genes, and bold colored lines indicate the mean expression trend of each cluster. Figure S4. Functional enrichment analysis of gene clusters derived from treatment-wise comparisons. (A–F) Bubble plots showing significantly enriched GO terms for the six major clusters identified from treatment-wise DEG clustering. Each panel corresponds to (A) Cluster 1, (B) Cluster 2, (C) Cluster 3, (D) Cluster 4, (E) Cluster 5, and (F) Cluster 6. Bubble size represents the number of DEGs assigned to each term, and color intensity indicates statistical significance (FDR < 0.05). Figure S5. Hierarchical clustering and expression profiles of DEGs from cultivar-wise comparisons. (A) Hierarchical clustering heatmap of DEGs identified from cultivar-wise comparisons, including C_AE vs. C_DH and I_AE vs. I_DH. Each row represents a gene, and the columns represent the two cultivar-wise pairwise comparisons. The color scale indicates log_2_ fold change, with red indicating positive values, green indicating negative values, and black indicating little or no change. (B) Expression profiles of the six major clusters. The *x*-axis represents the pairwise comparisons, and the *y*-axis represents log_2_ fold change. Gray lines represent the expression patterns of individual genes, and bold colored lines indicate the mean expression trend of each cluster. Figure S6. Validation of RNA-seq expression patterns by qRT-PCR. (A) qRT-PCR analysis of seven I_AE-enriched DEGs identified from the I_AE vs. I_DH comparison: ENSOPET00005000131, ENSOPET00005000137, ENSOPET00005002717, ENSOPET00005012956, ENSOPET00005012971, ENSOPET00005025345, and ENSOPET00005031318. (B) qRT-PCR analysis of five I_DH-enriched DEGs identified from the I_AE vs. I_DH comparison: ENSOPET00005009458, ENSOPET00005012618, ENSOPET00005013427, ENSOPET00005021531, and ENSOPET00005025498. Relative expression levels were normalized using *PoActin*, and data are presented as mean ± SD from three biological replicates. Dots indicate individual biological replicates. (C) Correlation between RNA-seq-derived and qRT-PCR-derived log_2_ fold-change values of the selected DEGs, showing general consistency between the two expression datasets. Table S1. Summary of RNA-Seq used in the study. Table S2. Primer sequences used for qRT-PCR validation of selected DEGs. Table S3. Differential expression and functional enrichment results for the four pairwise comparisons. (A) I_DH vs. C_DH, comparison between *P. tolaasii*-inoculated and mock-treated Dahyun. (B) I_AE vs. C_AE, comparison between *P. tolaasii*-inoculated and mock-treated Aenutari. (C) C_AE vs. C_DH, comparison between mock-treated Dahyun and Aenutari. (D) I_AE vs. I_DH, comparison between *P. tolaasii*-inoculated Dahyun and Aenutari. Table S4. Representative DEGs associated with cultivar-dependent transcriptional modules and selected for qRT-PCR validation.
