# Supplementary figures and images for: Comparative Transcriptomic Analysis Reveals Cultivar-Dependent Resistance Responses to Pseudomonas tolaasii in Pleurotus ostreatus
Source: J Fungi (Basel). 2026 Jul 17;12(7):525. doi: 10.3390/jof12070525 (PMC13412839; doi:10.3390/jof12070525)

Control

DH

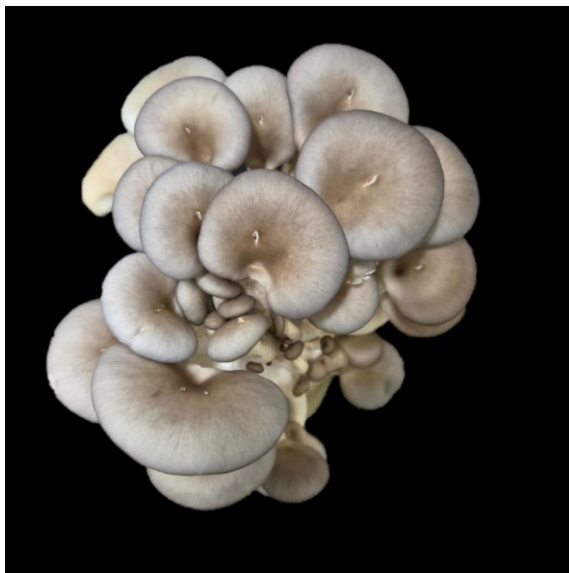

AE

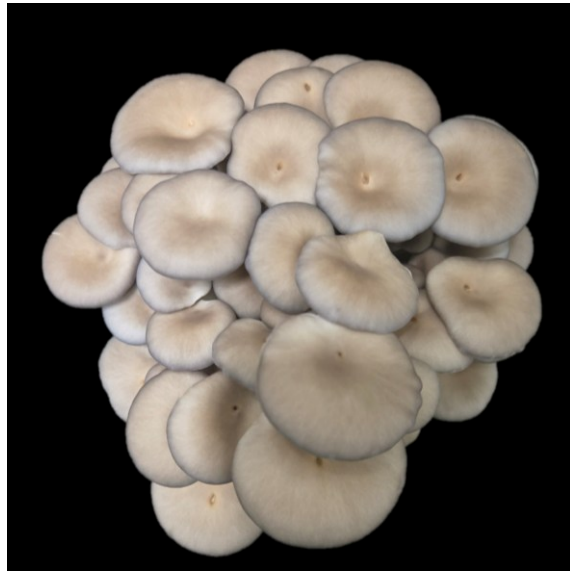

Inoculated

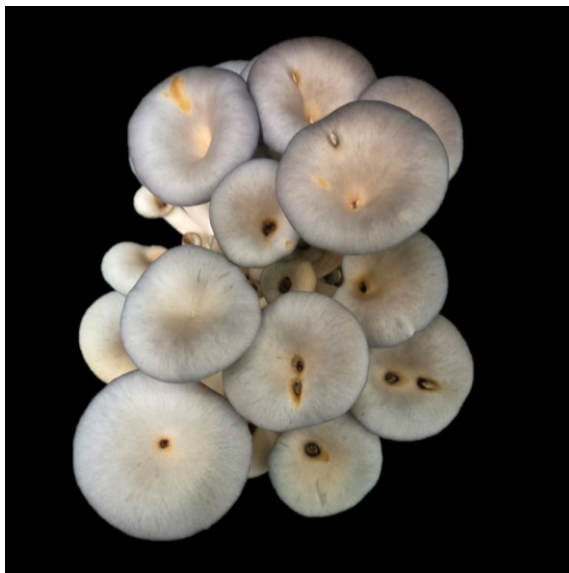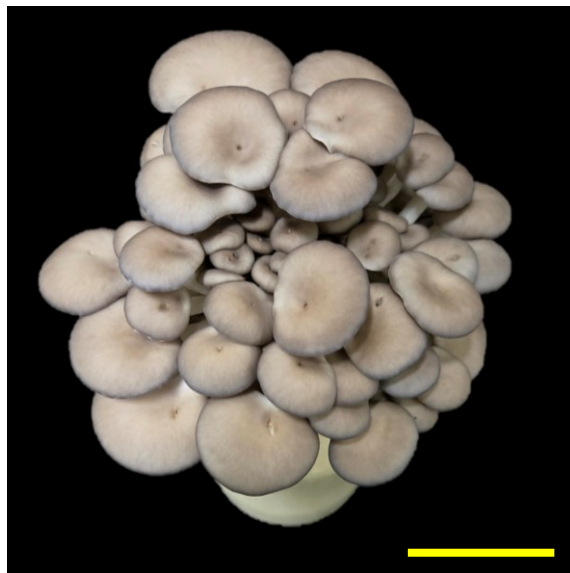

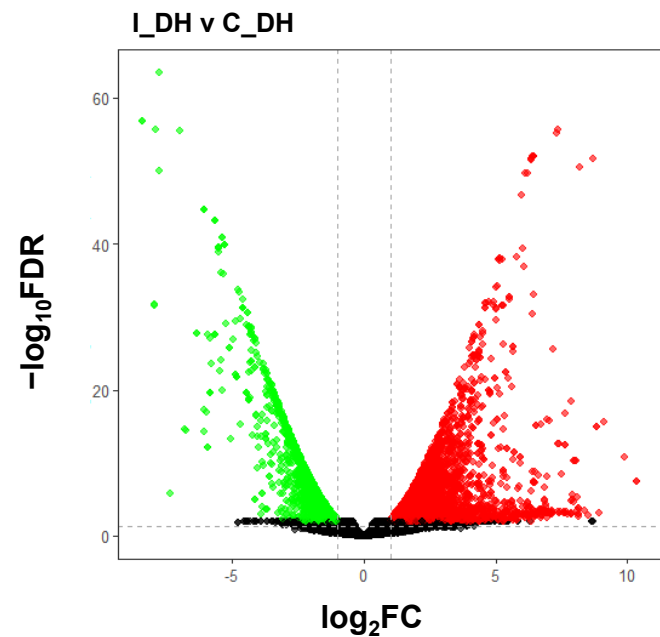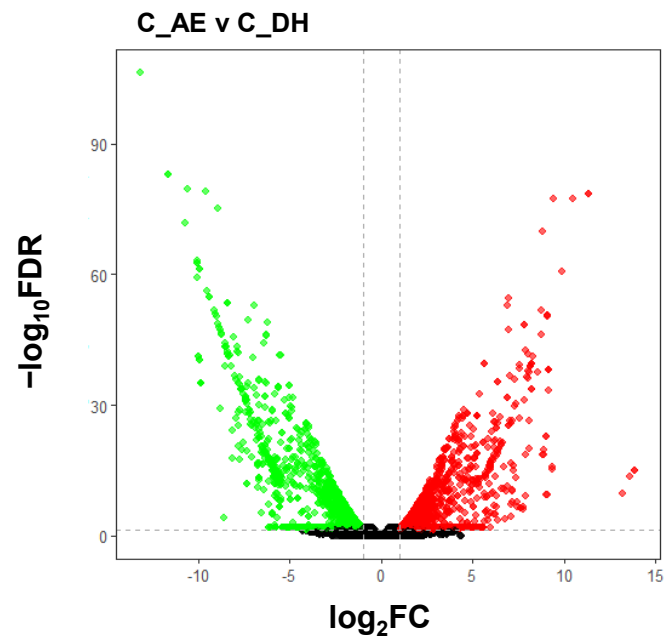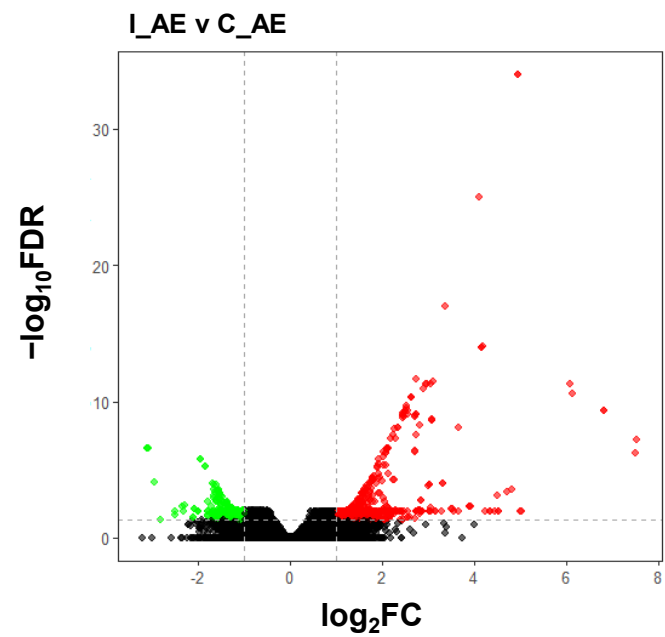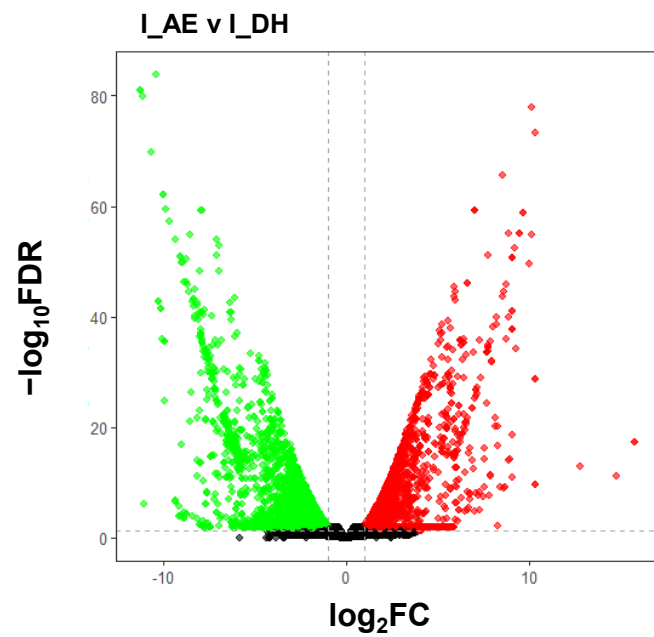

(A)

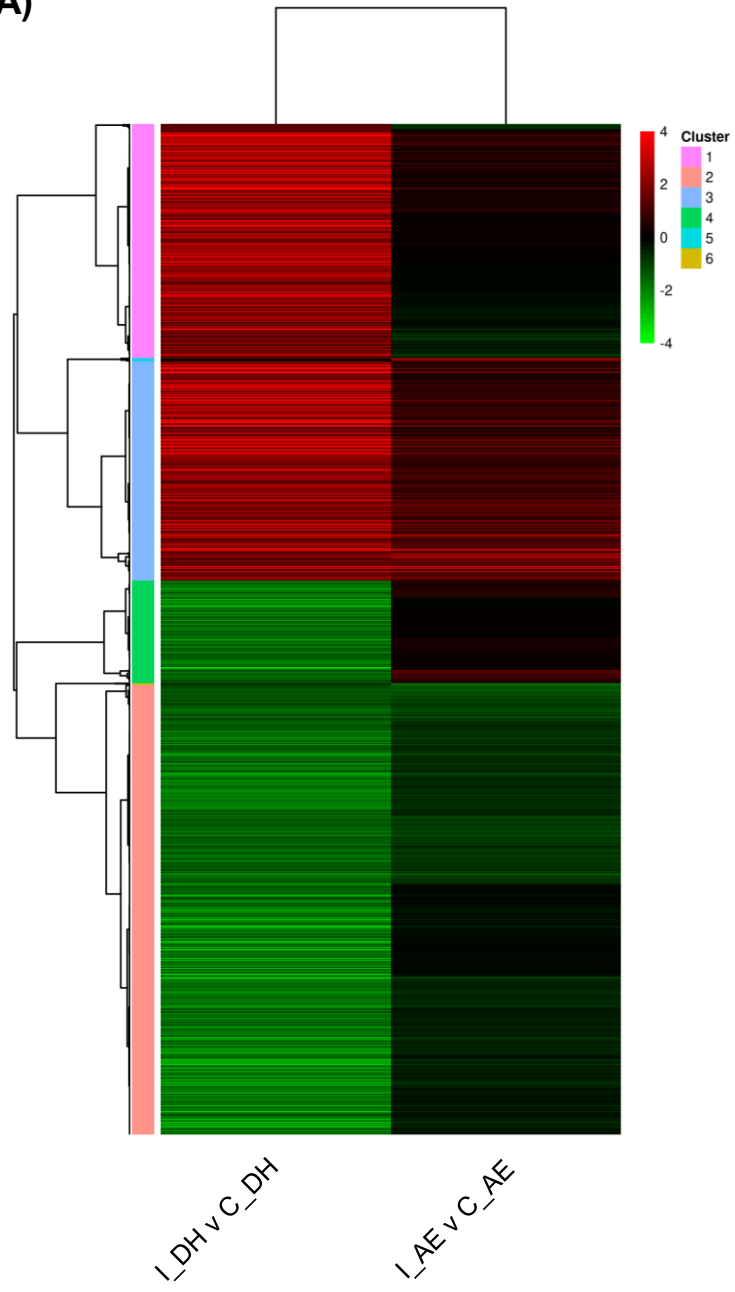

(B)

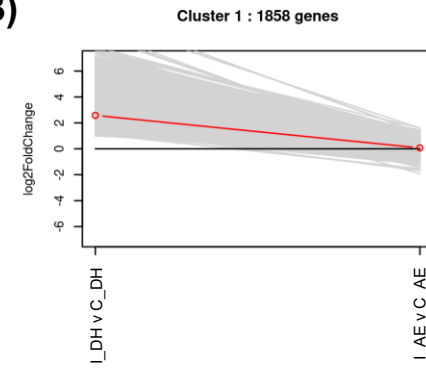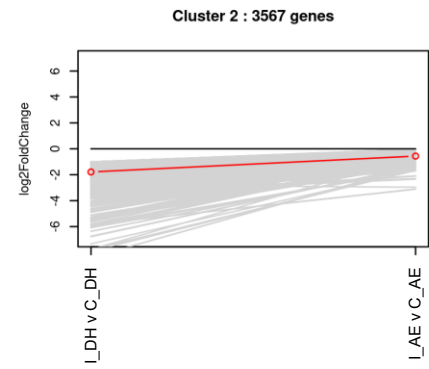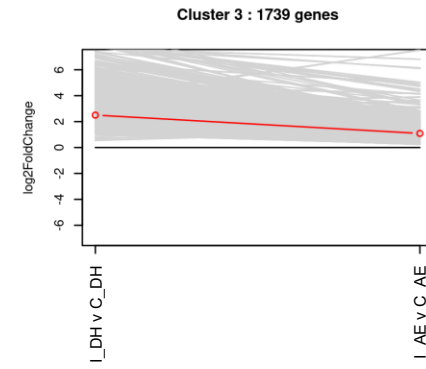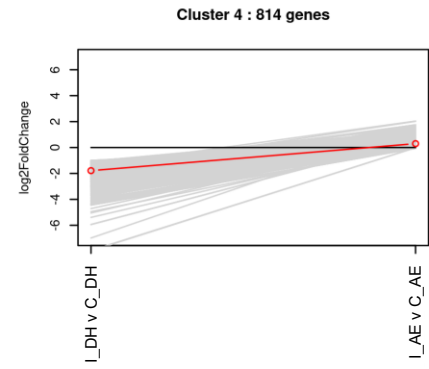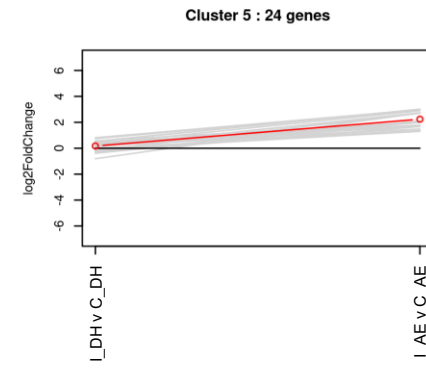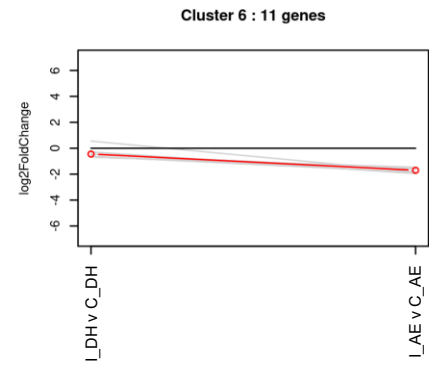

(A)

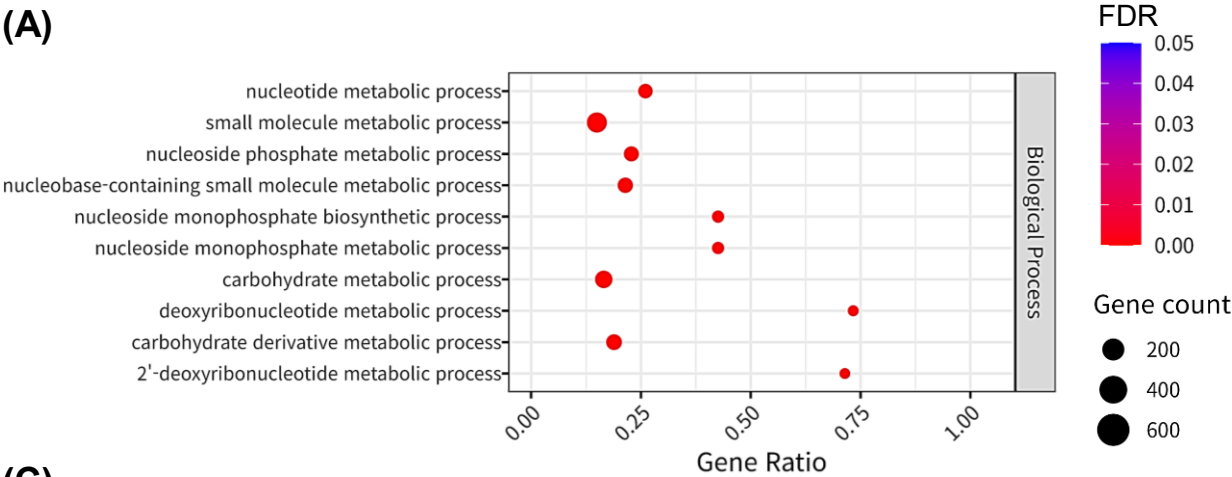

(B)

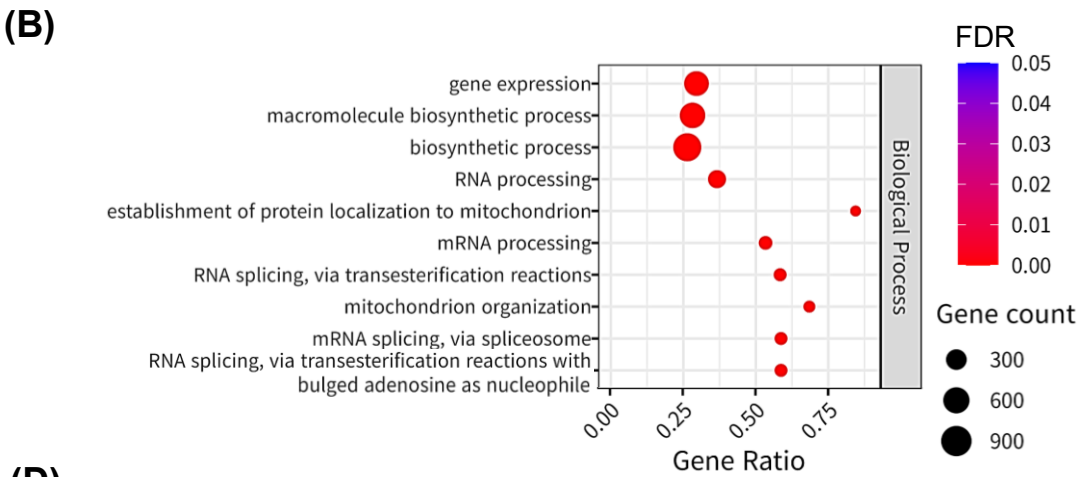

(C)

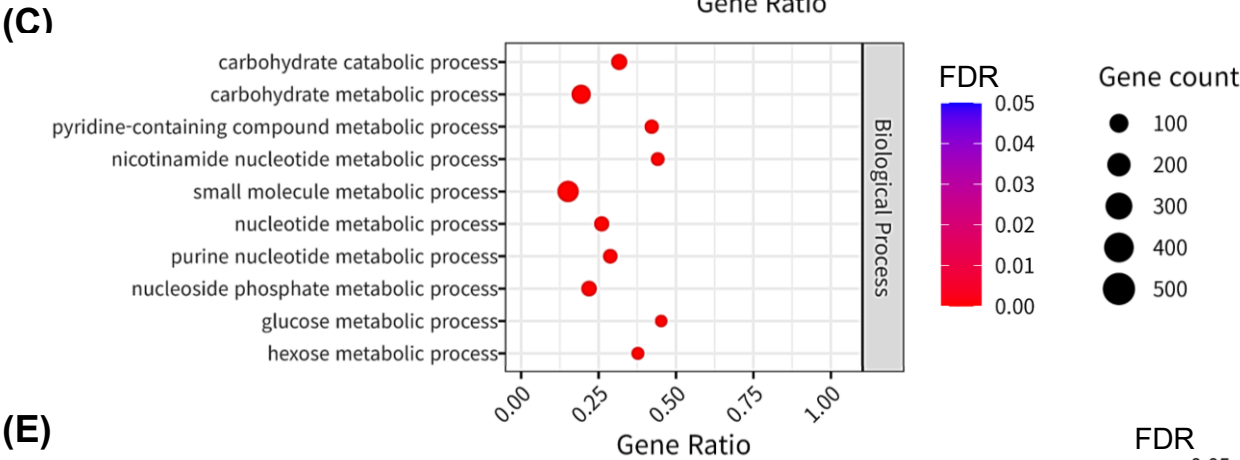

(D)

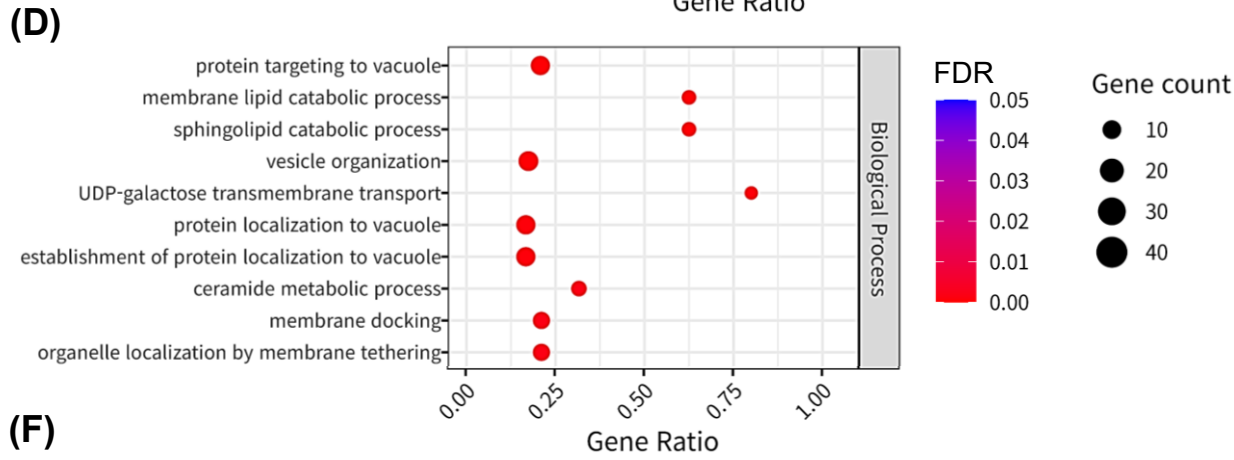

(E)

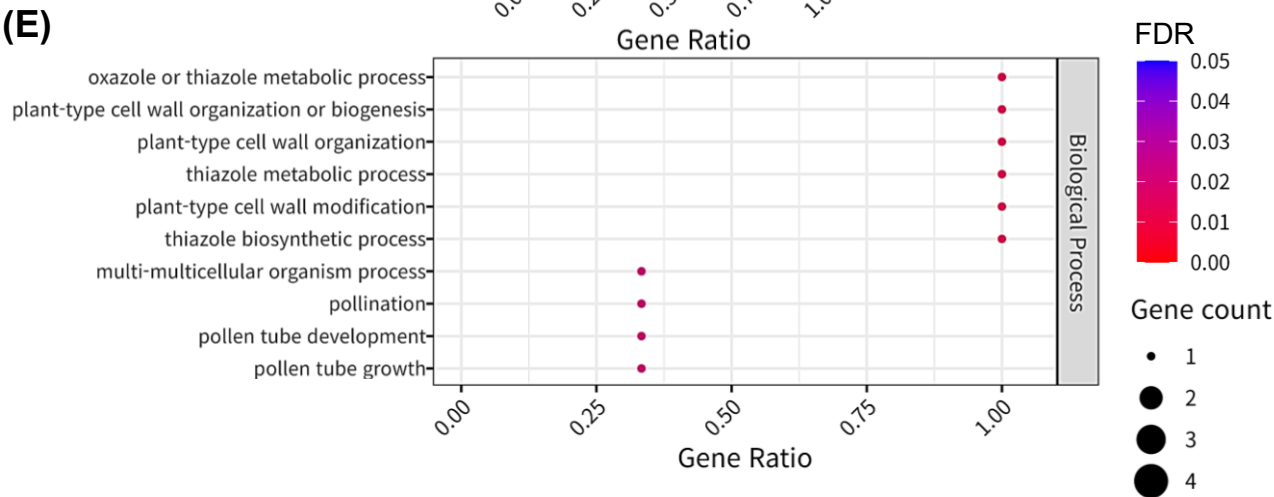

(F)

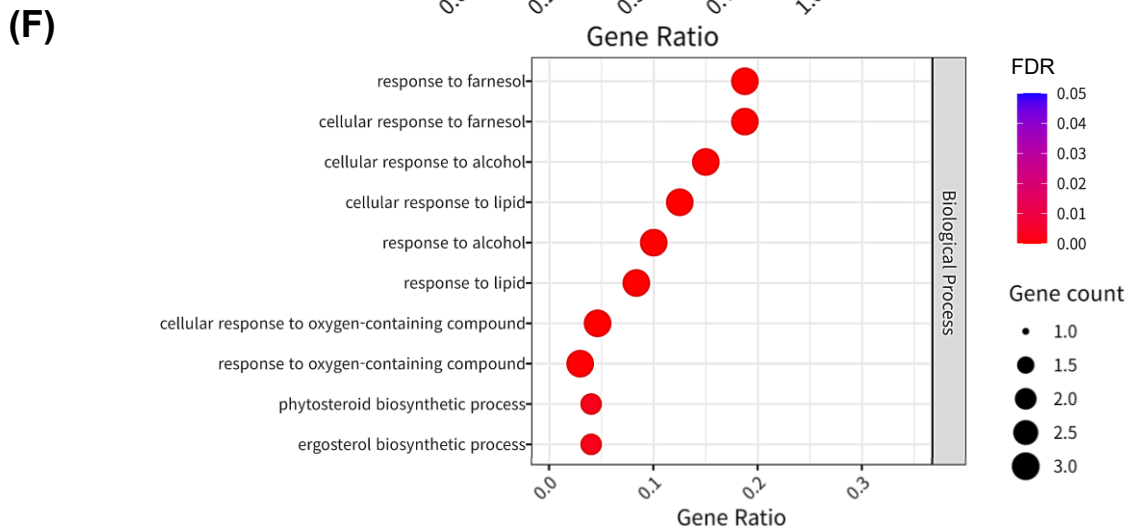

**(A)**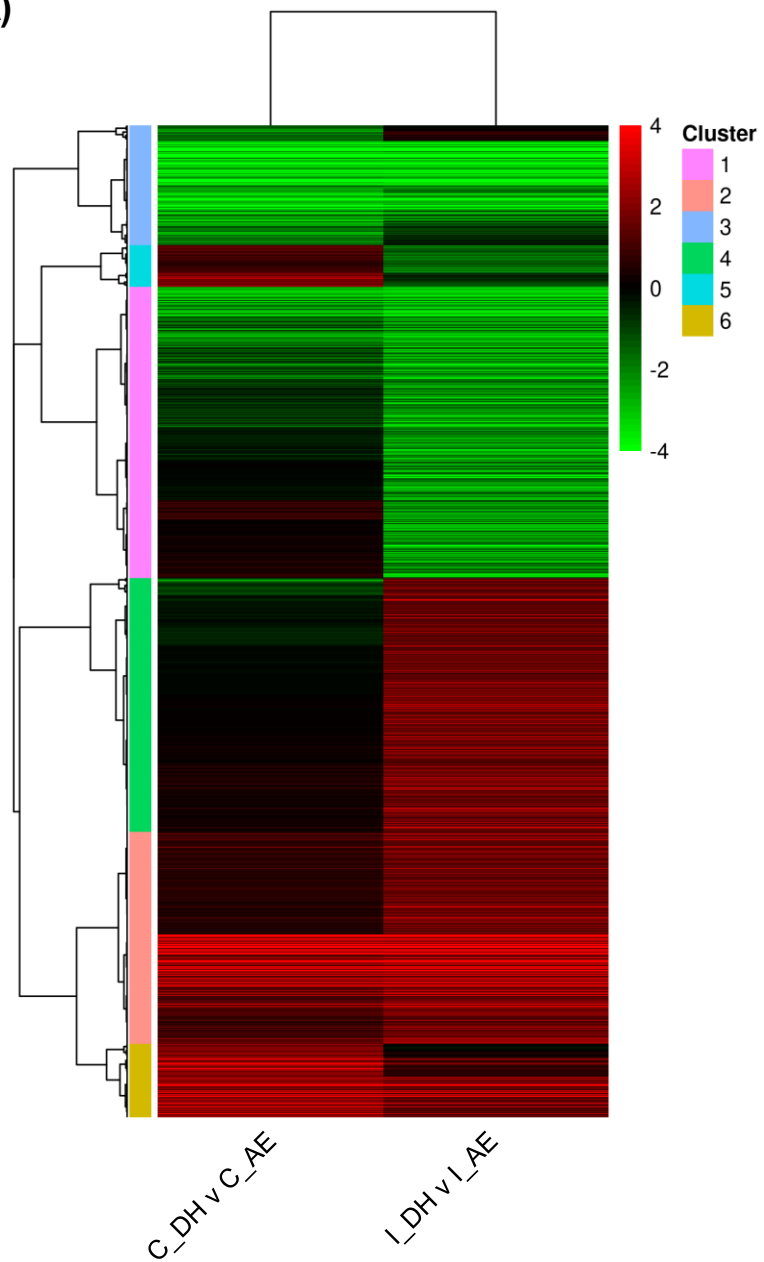**(B)**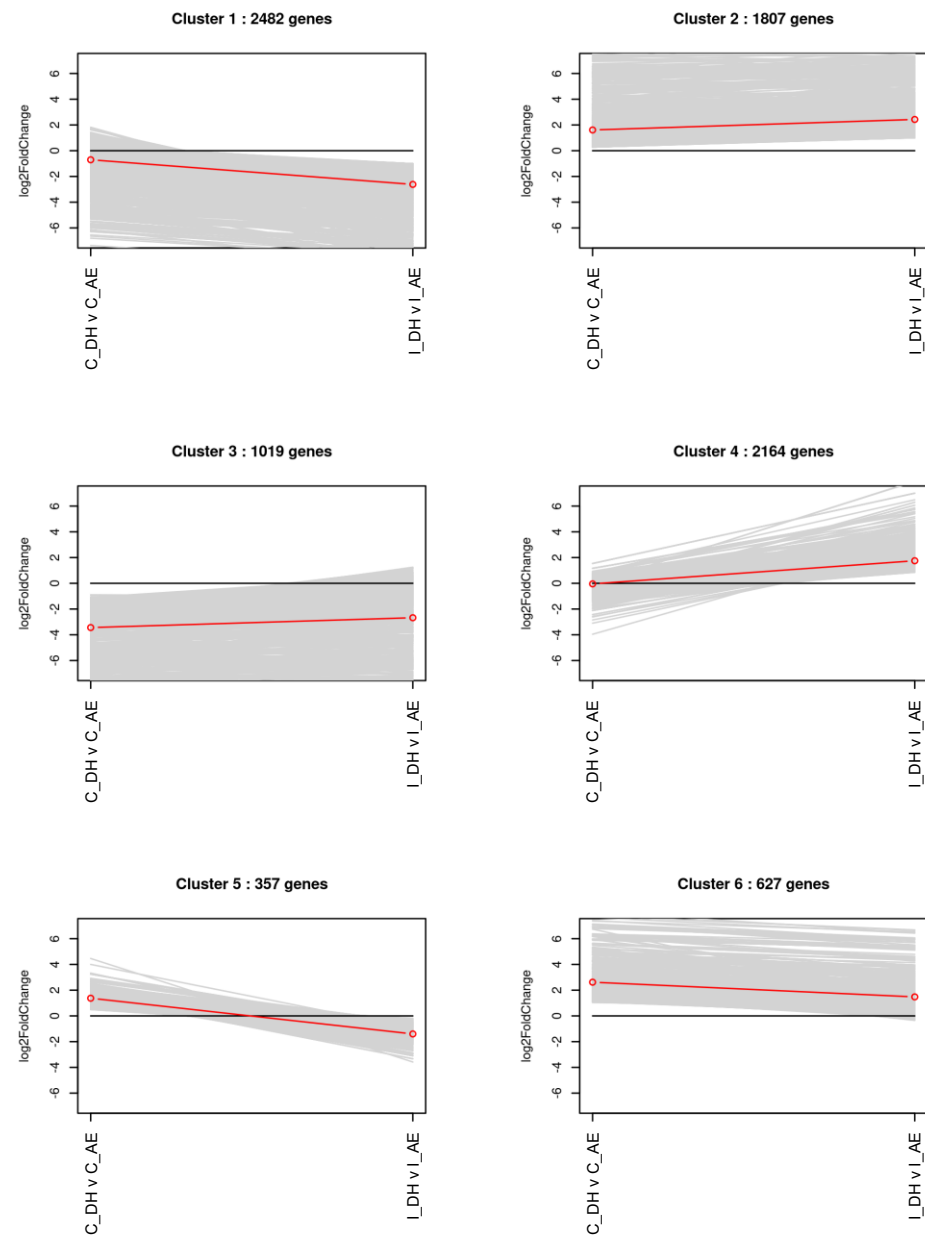

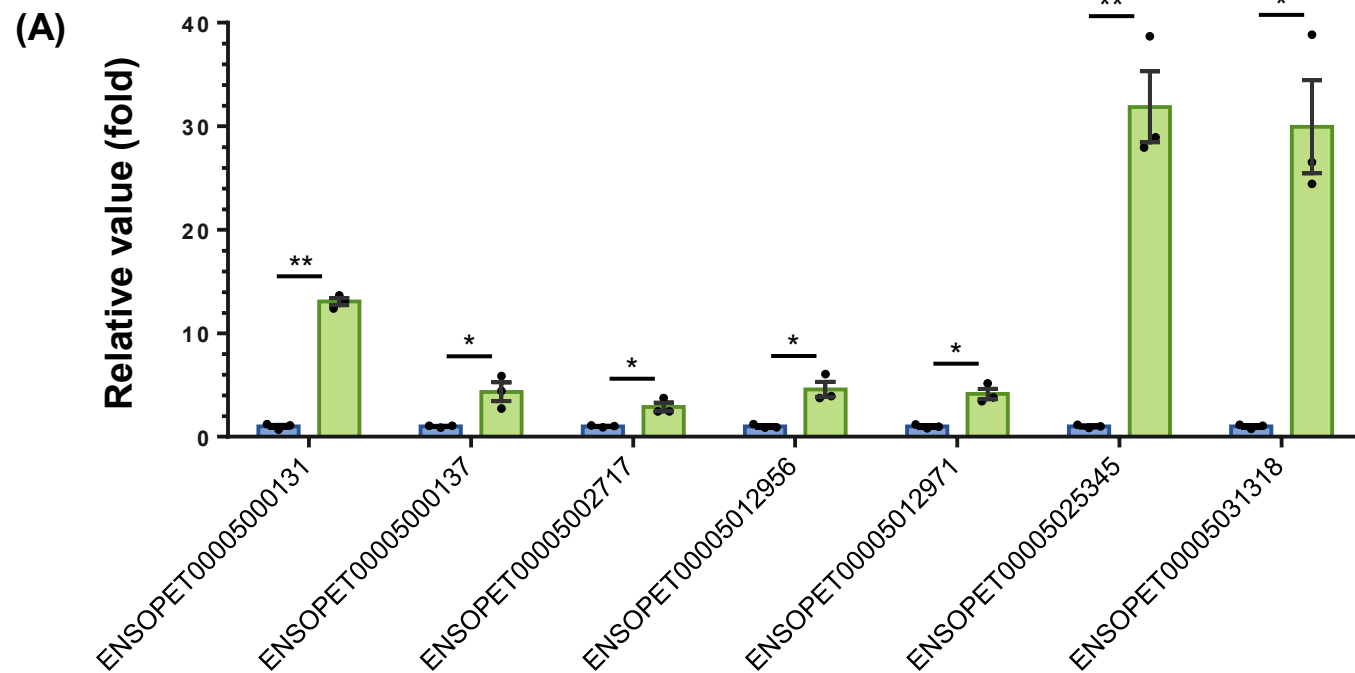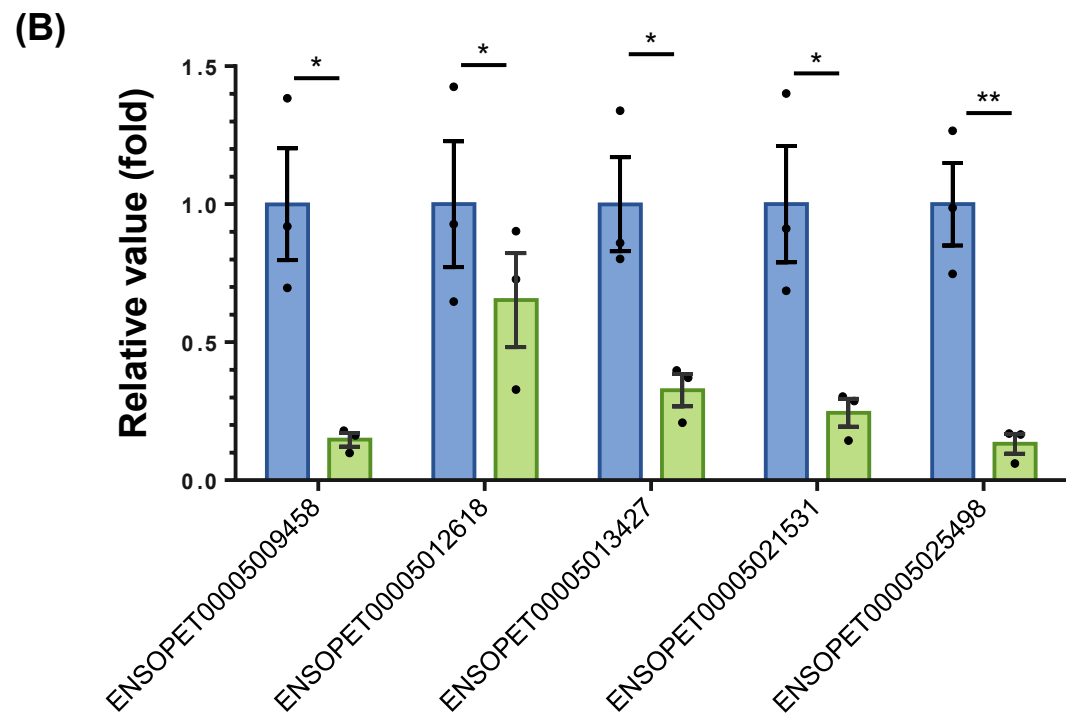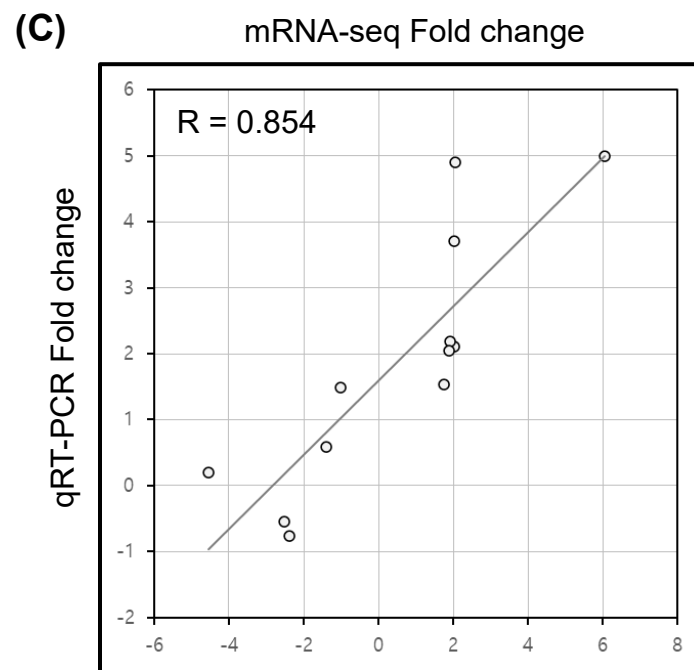

Supplement: Supplementary file 1 [file jof-12-00525-s001.zip › Supplementary Figure S1-S6_revised.pdf]
